# Colloidal spherical stibnite particles *via* high-temperature metallo-organic synthesis†

**DOI:** 10.1039/d4na00020j

**Published:** 2024-07-11

**Authors:** Maximilian Joschko, Christina Malsi, John Rapier, Paolo Scharmann, Sören Selve, Christina Graf

**Affiliations:** a Hochschule Darmstadt, University of Applied Sciences, Fachbereich Chemie- und Biotechnologie Stephanstr. 7 D-64295 Darmstadt Germany christina.graf@h-da.de; b Technische Universität Berlin, Zentraleinrichtung Elektronenmikroskopie (ZELMI) Straße des 17. Juni 135 D-10623 Berlin Germany

## Abstract

Antimony trisulfide (Sb_2_S_3_) is an emerging semiconductor with a high absorption coefficient and a bandgap in the visible range. This makes it a promising material for various electronic and optoelectronic applications. However, one of the main challenges is still the synthesis of the material, as it is usually obtained either as a nanomaterial in its amorphous form with inferior optical properties or in crystalline rod-like structures in the micrometer or sub-micrometer range, which leads to application-related difficulties such as clogging in inkjet printing or spraying processes or highly porous layers in film applications. In this study, a one-pot synthesis of highly crystalline, spherical Sb_2_S_3_ sub-micron particles is presented. The particles are growing encapsulated in a removable, wax-like matrix that is formed together with an intermediate from the precursors SbCl_3_ and l-cysteine. Both substances are insoluble in the reaction mixture but well-dispersable in the solvent 1-octadecene (ODE). The intermediate forms a complex crosslinked architecture whose basic building block consists of an Sb atom attached to three cysteine molecules *via* Sb–S bonds. Embedded in the matrix consisting of excess cysteine, ODE, and chlorine, the intermediate decomposes into amorphous Sb_2_S_3_ particles that crystallize as the reaction proceeds at 240 °C. The final particles are highly crystalline, spherical, and in the sub-micron range (420 ± 100 nm), making them ideal for further processing. The encapsulation method could not only provide a way to extend the size range of colloidal particles, but in the case of Sb_2_S_3_, this method circumvents the risk of carbonization of ligands or insufficient crystallization during the annealing of amorphous material.

## Abstract 2

Highly crystalline, spherical Sb_2_S_3_ particles of 420 ± 100 nm diameter are prepared using an *in situ*-forming matrix, preventing growth into large rods.

## Graphical abstract

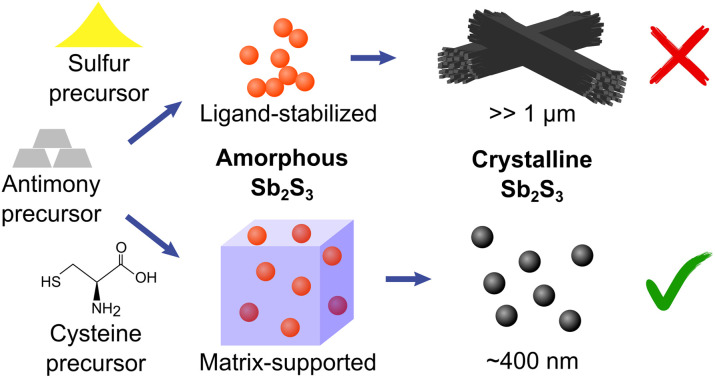

## Introduction

Semiconducting materials play an important role in modern society. They are used as memory chips,^1^ electronic components,^2^ sensors,^3^ for photocatalysis,^4^ in bioimaging,^5^ and many other applications. The demands on the properties of these materials are high: the ideal semiconductor has, in addition to its application-related properties, a high abundance, low toxicity, and is environmentally friendly.^6^ The interest in semiconducting Sb_2_S_3_ is growing for several electronic and optoelectronic applications, such as energy storage^7^ and optical data storage.^8^ However, the application most researchers are focusing on is the photovoltaic use of this material.^9–11^ This is due to the high absorption coefficient (1.8 × 10^5^ cm^−1^ at 450 nm) and direct bandgap energy around 1.7 eV of the crystalline phase.^12^

Electronic and optoelectronic examinations on Sb_2_S_3_ particles and Sb_2_S_3_ composite nanomaterials have shown promising results. For example, with voltammetry and impedance measurements, researchers have shown a high rate performance, cycle stability, and capacitance values for Sb_2_S_3_-carbon nanocompound electrodes.^13,14^ Other authors report photoelectric conversion efficiencies of up to 7.12% in performance tests of Sb_2_S_3_-based solar cells.^15,16^

Sb_2_S_3_ exists in two forms: an amorphous phase that appears orange-red and a crystalline, grayish-black phase (stibnite).^17^ However, the crystalline phase of semiconductors is usually preferred in application as the electronic properties are superior to the ones of the amorphous phase.^18^ Due to disorder effects and charge trapping, charge transport in amorphous materials is less efficient than in crystalline ones.^19^

So far, there are several strategies for synthesizing colloidal Sb_2_S_3_ particles: solvothermal,^20^ hydrothermal,^21^ and sonochemical methods,^22^ chemical bath deposition,^23^ and vapor deposition.^24^

Although similar chalcogenide nanoparticles, *e.g.*, Bi_2_S_3_, could be obtained in various sizes between 10 and 500 nm with a high crystallinity following comparable strategies,^25–27^ the results for Sb_2_S_3_ particles are different. While amorphous Sb_2_S_3_ nanoparticles are usually synthesized as spheres ranging from 20–30 nm in diameter to micron size with size deviations of about 15%,^28–30^ stibnite particles are usually received as elongated structures, *e.g.*, rods or urchin-like structures, with sizes in the micron or sub-micron range.^28,29,31–34^ This is the case because the particle formation starts with the amorphous phase and undergoes self-assembly and coalescence to larger particle sizes (>200 nm) until the crystallization begins.^29^

However, spherical particles have an advantage over rod-shaped ones regarding processing and applications. For example, they show favorable performance in inkjet printing due to an enhanced flowability^35^ and form more densely packed films after spin coating increasing conductivity.^36^ Though the preferred particle size is strongly dependent on the application, sub-micron- and nano-sized particles are demanded for, *e.g.*, inkjet printing or solar cells. Still, medium-sized crystals in the sub-micron range may lead to enhanced performance.^37–39^

To control particle aggregation, size, or shape, researchers developed several strategies. Most commonly, aggregation and size are controlled by electrostatic or steric stabilization, which can be achieved by adjusting the surface charge, adding ligands, or using coordinating solvents.^40–44^ In addition, ligands can affect the shape of the final particles.^45^ There are also a few reports in which researchers embedded precursors, which decompose upon heating or irradiation, in a polymer matrix to form a particle-polymer composite. The matrix also prevents aggregation and segregation of the particles.^46–48^ However, transferring these methods to the synthesis of crystalline Sb_2_S_3_ particles appears to be challenging.

Spherical stibnite particles are rarely reported in the literature because stibnite belongs to the orthorhombic crystal system and therefore prefers to grow in the direction of the *c*-axis into elongated, rod-like shapes.^49^ One approach was made by Zhu *et al.* using ZnS hollow spheres as a template for several metal sulfide hollow particles in a hydrothermal synthesis.^50^ The Sb_2_S_3_ spheres were about 1–2 μm in diameter. However, they reported annealing some of the metal sulfides after the synthesis. Thus, although the XRD revealed the stibnite structure for these particles, it remains unclear whether the initially formed particles were obtained in the amorphous or crystalline phase. Another approach was performed by Pan *et al. via* a hydrothermal synthesis using SbCl_3_, tartaric acid, and l-cysteine with a ratio of 2 : 12 : 3.^51^ After the reaction at 180 °C for 12 h in an autoclave, they received amorphous alveolate spheres of 2–3 μm in diameter which crystallized after annealing. Doing the same synthesis with a different ratio of the reactants (3 : 20 : 6), at a shorter time scale (8 h) and without annealing, Cao *et al.* claim to have obtained 2–3 μm sized crystalline Sb_2_S_3_ spheres with the same appearance as those obtained by Pan *et al.*^7^*via* an Sb-thioacetamide complex in a polystyrene solution, Sahoo *et al.* synthesized Sb_2_S_3_-carbon composite materials.^13^ By either stirring the solution for 2 h or using microwave irradiation for 10 min and post-annealing the intermediate products, they received Sb_2_S_3_ nanoparticles anchored on carbon sheets and carbon encapsulated Sb_2_S_3_ spheres, respectively. The spheres have a diameter of 600 ± 200 nm with a fully crystalline Sb_2_S_3_ core and a carbon shell. The nanoparticles on the carbon sheet are much smaller, about 5–10 nm, and mainly crystalline. However, some amorphous parts and some Sb_2_S_2_O are also present. Both materials show a good performance as electrode material.

In the following, a high-temperature synthesis that yields spherical stibnite particles with sub-micron size will be described. To achieve this result, l-cysteine and SbCl_3_ were dispersed in 1-octadecene (ODE). The cysteine acts not only as sulfur source but also forms a wax-like substance during the synthesis, which is insoluble in ODE due to the polar character of cysteine. The initially formed amorphous Sb_2_S_3_ particles are immobilized in this substance and, thus, prevented from growing into large, micron-sized rod-like structures upon crystallization. After the synthesis, the matrix can be removed and single stable particles are obtained. Furthermore, it will be shown that a stable intermediate substance consisting of antimony and cysteine is formed. This substance is crystalline, likely a highly crosslinked compound, and decomposes into Sb_2_S_3_ particles upon heating. The matrix is formed around this intermediate from excess cysteine, the solvent ODE, and the chlorine from SbCl_3_.

## Experimental section

Standard glassware was used for all experiments. Prior to the syntheses, all reaction vessels were cleaned with peroxymonosulfuric acid (Piranha solution), which was prepared by mixing sulfuric acid (95–97%, CHEMSOLUTE) with peroxide (30%, VWR chemicals) at a ratio of 7 : 1 (***Caution!****Piranha solution must be handled with extreme care as it is explosive and reacts violently with organic matter*). Subsequently, the glass vessels were rinsed with deionized (DI) water. The heating rate and reaction temperature were controlled using a temperature controller (LTR 3500; Juchheim Solingen).

### Materials

Antimony(iii) chloride (SbCl_3_, >99.95%) and isopropyl alcohol (IPA, 99.5%) were obtained from Sigma-Aldrich and ODE (90%, tech.) from Thermo Scientific. l-Cysteine (98%) was purchased from Carl Roth and *N*,*N*-dimethylformamide (DMF, for gas chromatography) from Supelco-Merck. All chemicals were used without further purification. The cysteine was thoroughly ground with a mortar before use.

### Synthesis

An argon atmosphere was maintained during all reaction steps.

First, 0.5 mmol SbCl_3_, 2.5 mmol l-cysteine, and 25 mL ODE were introduced into a 50 mL three-neck round-bottomed flask. To disperse the SbCl_3_ and the l-cysteine, the mixture was sonicated for approximately 15 min (Sonorex RK512H; 860 W; 35 kHz; Bandelin) until a homogenous, turbid dispersion was obtained. The reaction mixture was then magnetically stirred (800 rpm) at ambient temperature, and a vacuum (∼0.01 mbar) was applied for 30 min. The mixture was again set under argon and, subsequently, heated to 240 °C with a heating ramp of 10 K min^−1^. After holding the reaction temperature for 30 min, the reaction was stopped by removing the heating mantle.

### Particle purification

The nanoparticles were thoroughly purified by subsequent centrifugation and redispersing, and the matrix was removed during this process. Details of the procedure can be found in the ESI.†

### Characterization

#### Elemental analysis (CHNS/O analysis)

The samples were analyzed using a FlashEA 1112 CHNS/O Automatic Elemental Analyser from Thermo Scientific. For the CHNS analysis, approximately 2 mg of the sample was burned in a pure oxygen atmosphere over tungsten oxide at 1020 °C, and the evolving NO_*x*_ and SO_3_ were reduced over copper to N_2_ and SO_2_. The four components obtained, H_2_O, N_2_, SO_2_, and CO_2_, were chromatographically separated and detected *via* thermal conductivity detection.

The oxygen content was determined by immediately pyrolyzing approximately 2 mg of the sample in a helium atmosphere at 1060 °C. Organic oxygen is quantitatively converted to CO over nickel-containing carbon, chromatographically separated from other gases, and detected *via* thermal conductivity detection.

#### Energy-dispersive X-ray analysis (EDX)

To perform EDX analysis, the SEM was equipped with an EDAX X-ray detector (Octane Elect Plus). During the measurements, the SEM was operated with an acceleration voltage of 15 kV and a spot intensity of 50, while the working distance was set to 10 mm. The resolution of the detector was 126.2 eV.

The samples were deposited on copper grids in a layer several micrometers thick and measured on a STEM holder. Areas of 2 × 2 mm^2^ were measured at three different locations of the sample with 200 s accumulation time.

#### Atomic absorption spectroscopy (AAS)

An iCE 3000 spectrometer from Thermo Fisher Scientific was used for AAS. The samples were prepared by dissolving 10–15 mg substance in 0.5 mL conc. H_2_SO_4_ at 170 °C. A 0.1 mL aliquot was subsequently diluted with DI water to a total volume of 10 mL. The sample was vaporized in an acetylene flame.

#### Infrared spectroscopy (IR)

IR spectra were recorded with a JASCO FT/IR-4100. The measurement range was 4000–600 cm^−1^ with a step size of 4 cm^−1^. The samples were applied as slurry with IPA and left until dried or as a powder on the attenuated total reflectance (ATR) crystal.

#### Raman spectroscopy

Raman measurements were performed on a transmission spectrometer from Kaiser Optical (HL5R) equipped with a 514 nm argon laser and a CCD camera. Additionally, a Notch filter (514 nm) was used. The samples were measured for 200–240 s with 2–3 accumulations. A laser power of 1.1 mW was applied. The spectral resolution of the measurements is 5 cm^−1^. Due to grating changes, measurement artifacts occur at the wavelengths 2265 and 2324 nm.

#### Reflectance measurements

Since the particle dispersions were turbid, the UV/vis spectroscopy measurements were performed in reflectance mode. The instrument used was a Cary 5000 UV-Vis-NIR spectrometer (Agilent Technologies) equipped with an integrating sphere (internal DRA 2500). Dispersions consisting of IPA and 2–2.5 g particles per liter were measured in special quartz cuvettes (type 26.715/Q/10/Z20; Starna) in the range of 400 to 900 nm.

An artifact occurs at a wavelength of 800 nm due to a detector change of the spectrometer. The accuracy of the Tauc method was estimated on the basis of a study by Viezbicke *et al.*, who exemplarily evaluated 120 individual analyses of polycrystalline ZnO and found a deviation of about 0.03 eV.^52^

#### Mass spectrometry (MS)

To examine the samples, matrix-assisted laser desorption/ionization (MALDI; Autoflex speed TOF/TOF, Bruker Daltonik) and electron ionization (EI; Finnigan MAT 95, Thermo Fisher Scientific) techniques were used. For MALDI, the samples were ground with α-cyano-4-hydroxycinnamic acid (HCCA) at a ratio of 1 : 100. The instrument was equipped with a solid-state laser (2 kHz) and a time-of-flight (TOF) MS. Measurements are limited to a molecule size of ∼100 kDa. For EI, the samples were vacuum evaporated, ionized, and analyzed with a double-focusing sector field device with inverted Nier-Johnson geometry.

#### Scanning electron microscopy (SEM)

A Hitachi SU 5000 scanning electron microscope was used to record SEM images in the secondary electron (SE) mode with an electron acceleration voltage of 15 kV and a spot intensity of 30. The working distance was 3 mm. The particle dispersion (*c* = 1.5–2 g L^−1^) in water was dropped on a carbon-coated copper grid (carbon-coating type A, 6–10 nm thickness, Cu 400 mesh, Plano GmbH) and dried before the measurement. The software FIJI was used to evaluate the particle size for 200–300 particles per synthesis on multiple images.^53^

#### Transmission electron microscopy (TEM)

TEM investigations were conducted on a conventional Tecnai G^2^20 S-TWIN (FEI/TFS Company, USA) with LaB6 emitter, operated at 200 kV. For image acquisition, a US1000 CCD camera and DigitalMicrograph software (both: Gatan Inc., USA) were used. The sample preparation was identical to the preparation for SEM.

#### Thermogravimetric analysis (TGA)

The determination of organic residues in the final particle samples was performed using a Netzsch TG209 F1 libra with the external cooling system Julabo F32-MA. After a 10 min equilibration period, 4–8 mg dried sample was heated up to 300 °C at a rate of 10 K min^−1^ and held at this temperature for 50 min. A nitrogen gas flow of 20 mL min^−1^ was used during the measurement.

#### X-ray diffraction spectrometry (XRD)

The samples for XRD measurements were prepared as follows: the sample dispersions were drop-casted onto a glass slide (76 × 26 mm, Elka) at approx. 110 °C under constant argon flow until approx. 10 mg of the sample had dried on the glass slide. The samples were measured in Bragg–Brentano geometry with a fixed sample stage and movable X-ray source and detector arm. The instrument used was a Bruker D8 Eco equipped with a LYNXEYE XE-T detector and a Cu Kα1 radiation source (25 kV, 40 mA) with a radiation wavelength of 0.15405 nm. The angular range of the measurements was 20–80° 2*θ* with a step size of 0.025°.

#### X-ray photoelectron spectroscopy (XPS)

The spectrometer used for XPS measurements was from SPECS Surface Nano Analysis GmbH equipped with an Al/Mg X-ray source and a PHOIBOS 100 MCD energy analyzer. The powdered sample was pressed in indium foil before the analysis.

## Results and discussion

For this study, Sb_2_S_3_ particles were synthesized using the heat-up method. The precursors SbCl_3_ and L cysteine were used for this purpose. After SbCl_3_ and l-cysteine were dispersed in ODE, the heat-up process was started, and the reaction progress could be followed visually. Color changes in the reaction mixture at specific temperatures were associated with distinct reaction stages. Photographs of these stages can be found in Fig. S1 in the ESI.†

The first critical point in the synthesis was reaching a reaction temperature of 140 °C. The precursors, initially dispersed in the solvent, no longer form a milky white mixture (phase P0, Fig. S1(a)†) at this temperature. Instead, they aggregated, forming 1–3 large white flocs, which were slowly changing their color to pale yellow (PI, Fig. S1(b)†). As the reaction progressed, the flocs turned orange and began to break into smaller pieces at about 170 °C (PII, Fig. S1(c)†). They changed their color to dark red and sedimented at 200 °C (PIII, Fig. S1(d)†), where they finally turned black at 240 °C (PIV, Fig. S1(e)†). No further change was visible to the naked eye when the reaction continued at this temperature for 30 min. After the reaction mixture was cooled down at the end of the synthesis, a brownish-black product adhered to the walls of the reaction vessel.

The characterization of this brownish-black product is discussed first, and the individual reaction steps are examined in more detail in the following sections.

### Characterization of the reaction product

**Fig. 1:**
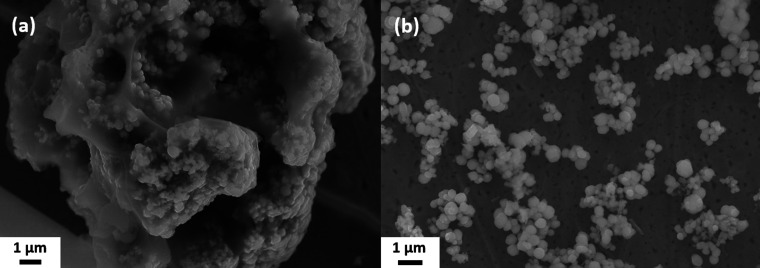
SEM images of the spherical Sb_2_S_3_ particles: (a) as received without cleaning and (b) after washing off the matrix.

The brownish-black synthesis product was examined by SEM after removing only the solvent ODE by washing once with hexane (Fig. 1(a)) and after washing several times with water/IPA and DMF (Fig. 1(b)). Both SEM images show spherical particles with an average diameter of 420 ± 100 nm. In the unpurified sample, the particles are immobilized in large lumps of a waxy matrix (Fig. 1(a)). The matrix can be removed in the purification process, yielding the bare particles, see Fig. 1(b). This finding is confirmed by performing a TGA (Fig. S2, ESI†). While the sample with the matrix around the particles showed a mass loss of about 1/3 of the initial mass, no mass loss was detected for the purified sample. However, an XPS measurement (Fig. S3 and S4, ESI†) suggests that ligands are still present on the particle surface. These data show that some ligands containing N and C. S and O are found as well. However, S is also present in the particles themselves, and O 1s overlaps with the Sb 3d_5/2_ peak. These findings suggest that the ligands are either cysteine or one of its decomposition products, considering the origin of the ligands. Based on several measurements, the overall accuracy of the TGA can be estimated at around 0.5–1%. Hence, the maximum content of organics in the sample is about 1 wt% corresponding to about 15 cysteine molecules per nm^2^. The particles are stable in aqueous dispersion, which was supported by the agreement of the calculated and observed sedimentation rates (2 cm d^−1^ for the lower size fraction).

**Fig. 2:**
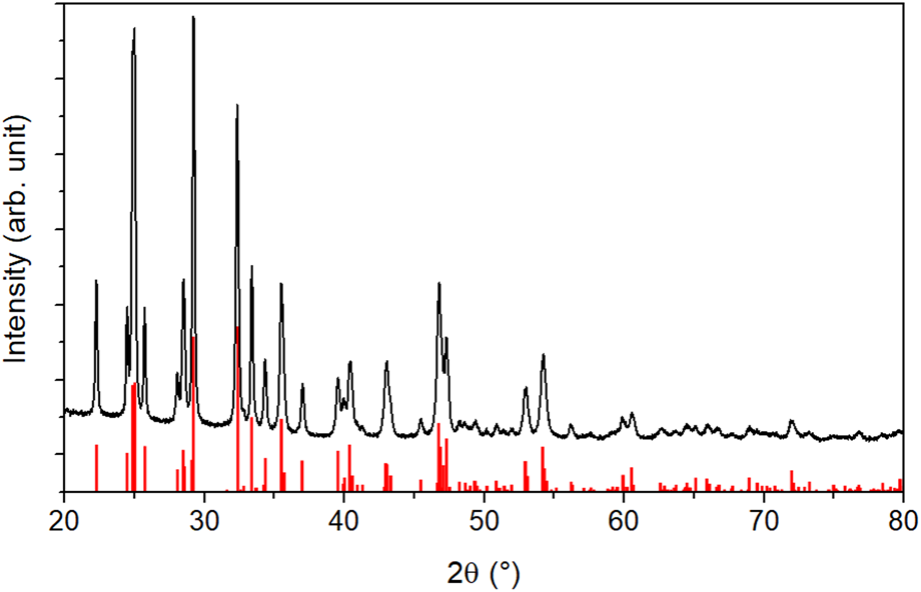
XRD of the purified particles. The pattern for stibnite (COD 9003460) is displayed in red.

An XRD analysis was performed to determine the crystallinity of the sample. The results shown in Fig. 2 reveal a highly crystalline material corresponding to the orthorhombic stibnite structure (COD 9003460). All diffraction peaks can be assigned to this pattern, indicating no impurities from, *e.g.*, antimony oxides.

Additionally, the crystallite size was estimated *via* the Debye–Scherrer equation (eqn (1)).^54^ The crystallite size *D*_*hkl*_ is calculated by dividing the product of the crystallite shape factor *K* and the wavelength *λ* by the width (full width at half maximum) of the diffraction peak *B*_*hkl*_ and the cosine of the corresponding Bragg angle *θ*.1
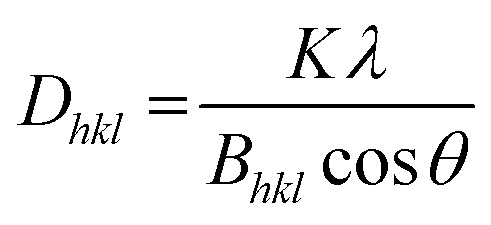


For different Laue indices, crystallite sizes between 40 and 55 nm were calculated. Thus, there is a clear discrepancy between the diameter estimated with the SEM, which provides no information about the crystalline composition of the particles, and the crystallite size estimated with the Debye–Scherrer equation, which only gives an average value.

**Fig. 3:**
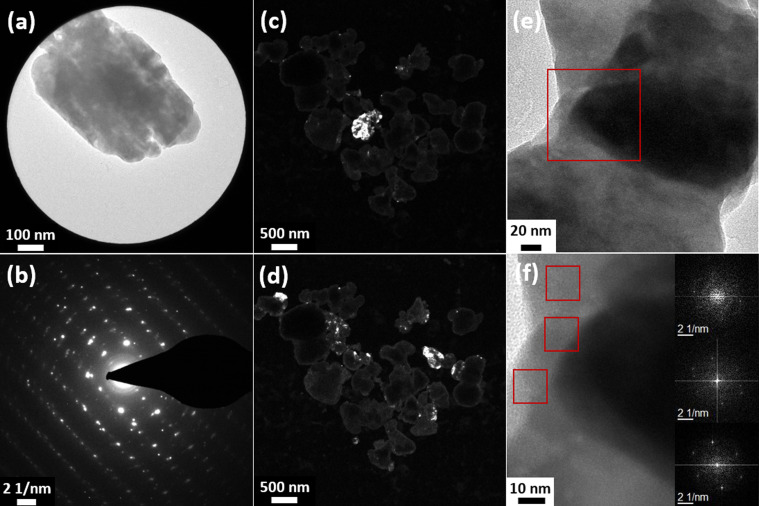
TEM analysis of the crystalline structure of the Sb_2_S_3_ particles: (a) TEM image of a particle with (b) the corresponding SAED pattern, (c) and (d) dark-field images of several particles showing different crystal planes in two different observation planes, and (e) and (f) HRTEM images of two crystallites in a particle. Image (f) is an enlarged view of the area in the red square in image (e). The insets in (f) show the FFT analyses of the three marked spots.

In order to unambiguously characterize the crystalline structure of the particles, measurements with different TEM techniques were performed, which are shown in Fig. 3 and S5 in the ESI.† The SAED analysis of a single particle (Fig. 3(a) and (b)) clearly shows a lattice structure, as would be expected from a single crystalline structure. However, some circular smearing effects can be seen in the diffraction images, which can only occur in samples containing crystalline areas with slightly different orientations or defects. Additionally, the SAED image of a different particle (Fig. S6(a) and (b), ESI†) was analyzed and the interplanar spacing was calculated. The values were compared with reference data (JCPDS card 42-1393) and the corresponding *hkl* indices were given (Table S2, ESI†). The calculated values match the reference data well.

In the dark-field images (Fig. 3(c) and (d)), different crystal planes appear bright depending on the observation plane/angle. The micrographs show that for certain diffraction spots, most areas of various particles appear in the same contrast, *i.e.*, have the same crystalline orientation. However, some smaller parts (approx. 50 nm), especially in the peripheral areas of the particles, show different crystal orientations. Based on these results, it can be concluded that the particles are mainly single crystalline but often contain some smaller, differently oriented crystallites in peripherical areas. Additional SAED and dark-field images of the same series can be found in Fig. S5 in the ESI.† As indicated by HRTEM micrographs in Fig. 3(e) and (f), the smaller crystallites are not aggregated to the large particles but rather intergrown with them. The images show two different crystallites in the same particle. The different crystal orientations are confirmed by Fast Fourier Transform (FFT) analysis (insets in Fig. 3(f)). Since the small crystallites are mainly found on the edges of the particles, it is likely that the crystal could not grow into its orthorhombic structure due to the surrounding matrix and, thus, crystal defects were formed or that other particles coalesced with already existing larger crystalline ones.

The optical properties of the particles were also investigated. The reflectance of the samples was measured, and subsequently, the Tauc plot was applied to determine the bandgap value. Before applying the plot, the reflectance data needs to be transformed into absorption data using the Kubelka–Munk equation (eqn (2)).^55^ The Kubelka–Munk function *F*(*R*_∞_) corresponds to the ratio of the absorption coefficient *k* and the scattering coefficient *s*. These coefficients, in turn, are correlated to the reflection of an infinitely thick specimen *R*_∞_.2
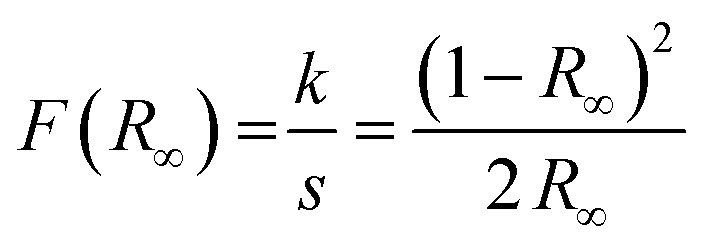


*F*(*R*_∞_) replaces the absorption coefficient *α* in the Tauc equation, which is displayed in eqn (3).^56,57^*α* is expressed by the Planck constant *h*, the photon frequency *ν*, the transition factor *γ*, the bandgap energy *E*_g_, and a constant *B* described by Davis and Mott as magnitude of the optical absorption constant.

3 *αhν*^1/*γ*^ =*B*(*hν* − *E*_g_)

*γ* describes the type of the bandgap transition. It equals 1/2 for a direct allowed transition and 2 for an indirect allowed transition.

There are different opinions in the literature on whether the bandgap of crystalline Sb_2_S_3_ is direct^58–60^ or indirect.^61–63^ Still, the bandgap is mainly located between 1.6 and 1.8 eV, with the indirect bandgap about 0.1 eV lower than the direct one. Theoretical calculations have shown that the indirect transition is energetically more favorable.^64,65^ Since the difference to the direct transition is small, the authors conclude that the direct transition is most likely dominant.

**Fig. 4:**
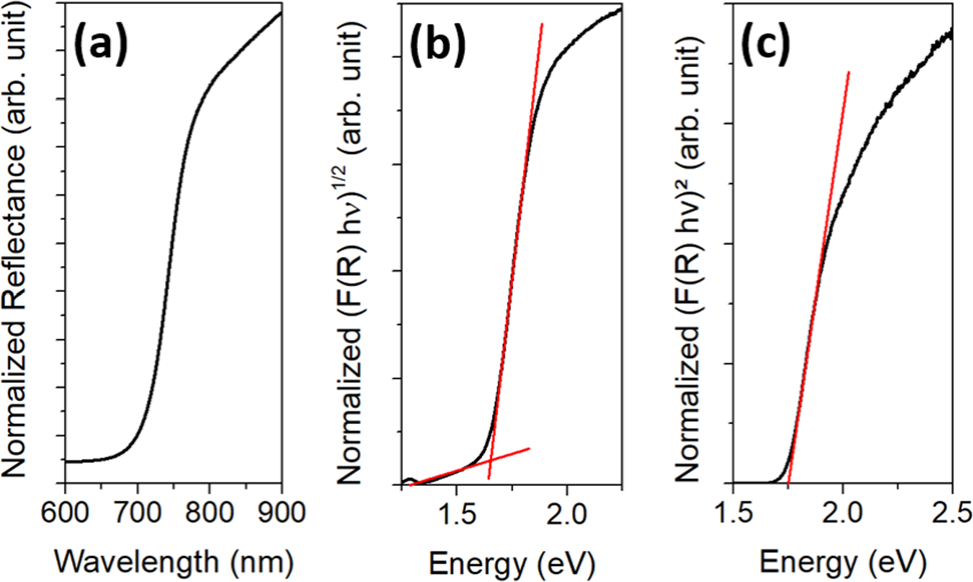
UV/vis data of the final particles showing (a) the measured reflectance data, (b) the Tauc plot for estimating the indirect bandgap, and (c) the Tauc plot for estimating the direct bandgap.

Fig. 4(a) shows the measured reflectance data, while Fig. 4(b) and (c) show the data transformed according to the Tauc equation for an indirect and a direct bandgap transition, respectively. The reflectance curve shows one steep slope, as is usual for a phase-pure semiconductor, around 720 nm. To estimate the bandgap of the material, a tangent is drawn to the slopes of the Tauc plots, and the *x*-value is taken where the tangent intersects the extended baseline of the plot. The results correspond to 1.65 eV for the indirect bandgap and 1.75 eV for the direct bandgap.

### Formation and growth of the Sb_2_S_3_ particles

The results shown in the last section strongly suggest that the matrix is the critical component in the formation of colloidal spherical stibnite particles. Hence, the reaction process was thoroughly studied, and samples were taken at different reaction stages. These samples, taken at the temperatures 140 °C (PI), 170 °C (PII), 200 °C (PIII), and 240 °C (PIV), were further characterized by SEM, XRD, EDX and CHNS/O analysis, UV-vis, and IR spectroscopy.

**Fig. 5:**
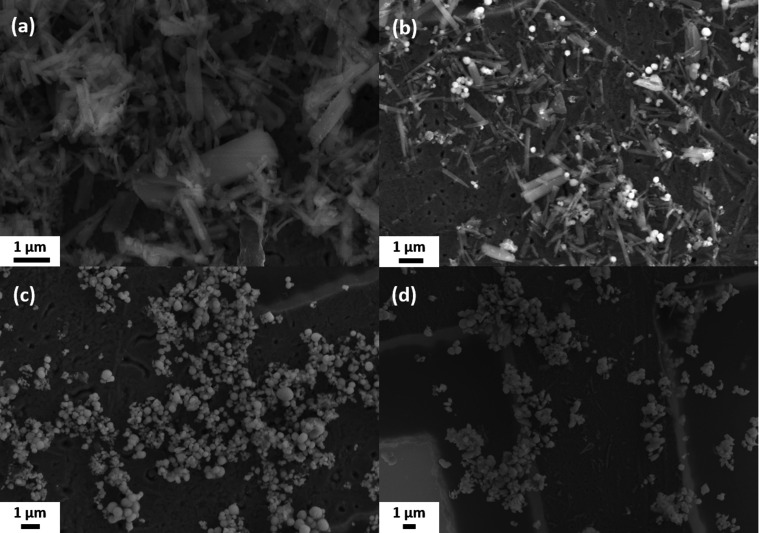
SEM images of the different reaction stages of the synthesis: (a) after 140 °C (PI), (b) after 170 °C (PII), (c) after 200 °C (PIII), and (d) after 240 °C (PIV). The samples were purified with the same procedure as the final particles.

The samples were purified by the same procedure as the final product prior to characterization (see Experimental part for details). Fig. 5 shows SEM images of the different samples. The samples PI (Fig. 5(a)) and PII (Fig. 5(b)) contain rod-shaped particles with a length of a few micrometers. However, in sample PII, small spherical particles with a diameter of 220 ± 60 nm are also found. In sample PIII, these particles are still present but have grown larger (diameter of 280 ± 110 nm), while the rod-shaped particles are no longer found (Fig. 5(c)). In sample PIV (Fig. 5(d)), the spherical particles have become even larger with a diameter of 430 ± 110 nm and do not differ significantly in size or appearance from the final product. Statistical size distributions of these samples can be found in the ESI in Fig. S7.†

**Fig. 6:**
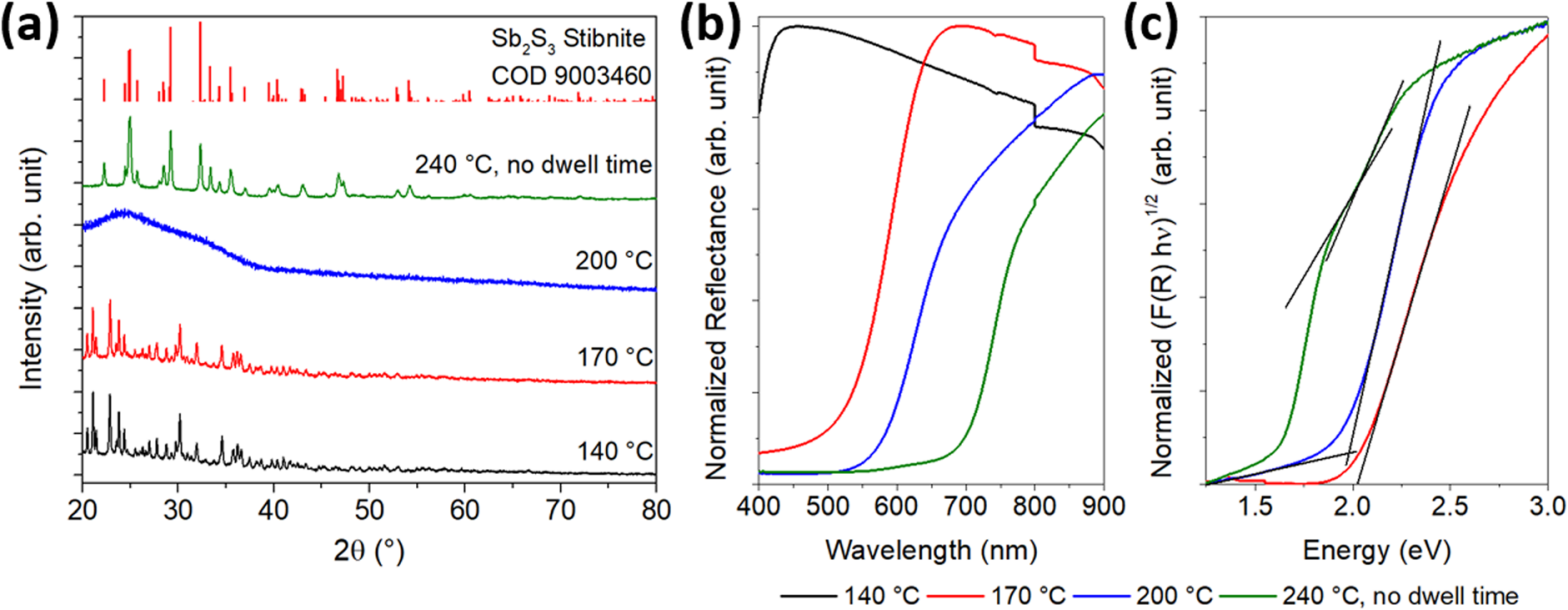
Phase and optical characterization of reaction intermediates at different reaction stages of the synthesis. XRD analysis (a) and reflectance measurements (b) were performed using samples taken at 140 °C, 170 °C, 200 °C, and 240 °C (without 30 min additional reaction time). The Tauc plot (c) was applied to all reflection data except that of the sample taken at 140 °C, since no band gap transition can be seen.

In addition, the growth process of the particles was characterized crystallographically and optically. The results of the XRD analysis and the UV/Vis spectroscopy are shown in Fig. 6. XRD (Fig. 6(a)) revealed a highly crystalline structure for the samples PI and PII, which was neither be found in the COD database nor corresponded to l-cysteine or SbCl_3_ (reference diffractograms in Fig. S8, ESI†). This suggests that a new substance was formed at 140 °C. Since no other phases are detected in sample PII, the small spherical particles likely correspond to the amorphous phase of Sb_2_S_3_. The reflectance measurement (Fig. 6(b)) shows that no semiconducting substance was formed in PI, and the Tauc plot (Fig. 6(c)) gives further indication that the particles in PII consist of amorphous Sb_2_S_3_, which has a bandgap value between 2.0 and 2.2 eV.^29^ For the particles of sample PII, a band gap value of 2.02 eV was determined. To obtain further information about the materials formed during the reaction process, the purified solids were characterized by CHNS/O analysis and EDX. Table 1 provides additional information on the elemental (atomic) composition of the samples and the sizes of the spherical particles.

**Table 1:** Elemental (atomic) composition of the purified solids of the different reaction stages, the diameter of the Sb_2_S_3_ particles, and their bandgap values

Sample	PI	PII	PIII	PIV	Final
Temp. (°C)	140	170	200	240	240 (30 min)
Sb	1.0	1.6	2.0	2.0	2.0
S	2.9	3.9	3.0	3.0	3.0
C	9.0	9.0	—	—	—
H	19.5	19.5	—	—	—
N	2.8	2.9	—	—	—
O	6.6	6.6	—	—	—
Diameter (nm)	**—**	220 ± 60	280 ± 110	430 ± 110	420 ± 100
Bandgap (eV)	**—**	2.02	1.99	2.01/1.75	1.75

The white crystals in PI are composed of antimony and elements normally found in organic molecules, almost corresponding to the stoichiometry of cysteine (C_3_H_7_N_S_O_2_). Chlorine, however, was not found. The results for this substance are discussed in more detail in the following section. Since the particles in PII are Sb_2_S_3_, their composition results from a mixture of the substance from PI and Sb_2_S_3_ in an atomic ratio of 3 : 2 for the element antimony. Along with the white rod-like particles, the crystalline XRD pattern disappeared in sample PIII. Also, only the elements antimony and sulfur remain in a 2 : 3 ratio. These results, along with a band gap value of 1.99 eV for this substance, indicate that the white substance is likely an intermediate in the formation of Sb_2_S_3_ particles, which are in an amorphous state up to this point. Compared to PII, the particles slightly increased in diameter.

For sample PIV, the X-ray diffractogram and optical data are changed compared to those of the samples of earlier reaction stages. A crystalline pattern corresponding to the stibnite structure (COD 9003460) can be seen in the diffractogram, and the absorption edge is shifted to higher wavelengths in the UV/vis spectrum. However, a second absorption edge similar to that of the amorphous material is present. After applying the Tauc plot, this sample fraction becomes more visible. Thus, two band gap values of 2.01 and 1.75 eV could be determined corresponding to the bandgaps of amorphous and crystalline Sb_2_S_3_, respectively. Apparently, the particles are not yet fully crystallized at 240 °C, so the temperature must be held at this level for several minutes to allow a complete conversion. The crystallite sizes, derived from the Debye–Scherrer equation for different Laue indices, were about 35–50 nm, which is about 5 nm smaller than those of the final particles after 30 min reaction time at 240 °C. The particle diameter obtained from SEM, however, is identical to that at the end of the reaction, but with a diameter of more than 400 nm, they are significantly larger than the amorphous particles.

### Characterization of the reaction intermediate

The results discussed in the last section suggest that there is an intermediate in the formation of the colloidal Sb_2_S_3_ particles. This intermediate is a white, crystalline substance with an atomic ratio of elements that almost corresponds to a composition of antimony and cysteine in a ratio of 1 : 3. The main difference to the atomic ratio of the elements in cysteine is that the value for hydrogen is decreased (∼0.5 atoms per theoretical cysteine molecule) and the value for oxygen is increased (∼0.2 atoms per theoretical cysteine molecule). Assuming that the sample contains some water, *e.g.*, from the purification process or from handling in an ambient atmosphere, the basic building block of the intermediate could be Sb(C_3_H_6_NSO_2_)_3_·0.75H_2_O.

To investigate the chemical structure of this substance further, Raman and IR spectroscopy, as well as an MS analysis, were performed. The results are shown in the ESI Fig. S9 and S10.† Comparing the IR and Raman spectra of sample PI (Fig. S9, ESI†) with the reference spectra of l-cysteine and l-cystine, the similarity is low for the spectra of cysteine but high for the spectra of cystine. In particular, the absence of the –SH vibration in the measured Raman spectrum should be noted. However, there is also a clear difference in the Raman spectrum between the measured spectrum and that of cystine. The strong signal at ∼500 cm^−1^ assigned to the vibrational mode of the disulfide bond in cystine,^66^ is not present. Instead, an intense mode at ∼355 cm^−1^ is found. According to several reports, Sb–S stretching vibrations are found around these wavenumbers.^67,68^

For the MS analysis, MALDI and EI were used to ionize the sample as the substance is not soluble in common solvents such as water or isopropanol. With MALDI, two molecular patterns are visible in the spectrum, around 496.9 u and 707.9 u. However, these patterns suggest molecules consisting of one antimony atom, at most one sulfur atom, and mainly cysteine residues with no sulfur but an increased amount of oxygen. Since this is inconsistent with the elemental analysis, the molecule is assumed to be a highly crosslinked polymer with a molecular weight greater than 100 kDa, which is above the mass limit of MALDI for polymeric species.^69^ The molecular patterns found could be due to a slight degradation by oxygen.

With EI, in addition to several Sb_*x*_ and Sb_*x*_O_*y*_ fragments, the fragmentation pattern shown in Fig. S10(a)† was obtained. The pattern has similarities to both the cysteine and cystine patterns (Fig. S10(b) and (c),† respectively), indicating that the structure is probably intermediate between the two.

In summary, the intermediate substance is assumed to have the following properties:

1. General composition of Sb(C_3_H_6_NSO_2_)_3_.

2. Structure of the organic part in between cysteine and cystine.

3. –C–S and –Sb–S bonds but no –S–H or –S–S bonds.

4. Large, crosslinked molecule with *M*_w_ > 100 kDa.

5. No absorption in the visible spectrum.

There are also several reports of Sb(iii) covalently bound to organic molecules and, additionally, complexed by other molecules, resulting in a 4- or 5-fold coordinated structure.^70–72^ Thus, a molecule would be conceivable in which Sb(iii) is covalently bound to the S atom of three cysteine molecules and coordinated to the carboxyl group and/or to the amino group of one or two other cysteine molecules. The coordinative bonds would also lead to crosslinking. However, further analysis is needed to determine the exact structure of this molecule.

### Matrix analysis

The matrix appears to prevent the amorphous particles from growing into large (micron-sized) rods during crystallization, so they tend to crystallize into a spherical shape. For this reason, the composition of the matrix was studied in more detail. Table 2 shows the results of the CHNS/O analysis, AAS, and EDX of non-purified samples at 140 °C (MI), 170 °C (MII), and at the end of the synthesis (Mfinal). Non-purified means that the samples were only washed with hexane to remove the ODE but not the matrix.

**Table 2:** Elemental (atomic) composition of three samples at different reaction stages, including the matrix

Sample	MI	MII	Mfinal
Temp. (°C)	140	170	240, 30 min
Sb	1.0	1.0	1.0
S	7.4	6.6	2.0
C	33.7	35.0	8.1
H	73.8	77.5	15.4
N	7.4	7.5	3.3
O	14.2	13.4	1.1
Cl	2.3	2.8	2.9

It can easily be seen that the Sb content of MI with respect to the organic moieties is less than half of that of PI. In addition, chlorine is present in the sample, and the stoichiometry of the organic moiety does not resemble cysteine. However, the ratio of S : N : O in the matrix is almost the same as that of cysteine. This result indicates that the increased C and H content originates from the ODE. This finding can also be correlated with the ratio of the excess amount of these two elements, which is 1 : 2; the same ratio as for ODE. It is assumed that the ODE molecules are embedded in the forming matrix, but a further reaction of them cannot be excluded.

It can also be seen that, in contrast to the sample PI, no excess oxygen can be found with respect to sulfur/nitrogen to match the ratio in cysteine. Furthermore, the Sb : S ratio (7.4 : 1) is lower than the ratio at the beginning of the synthesis (1 : 5). This is another indication that the excess H and O atoms of PI originate from washing with water. It can be further concluded that some antimony is lost during the purification process.

As the reaction progresses, the amounts of S and O decrease, while the amounts of C, H, and Cl increase slightly relative to Sb. The decrease in S and O indicates the beginning of cysteine decomposition. However, the amino group seems to belong to a solid rather than a gaseous decomposition product because the amount of N increases relatively to C, H, S, and O. Variations in C and H could be explained by slight differences in the embedding of the ODE into the matrix or an ongoing reaction with it.

The ratio of Sb and Cl is close to their ratio in SbCl_3_. It is assumed that not all SbCl_3_ has reacted at 140 °C. Since SbCl_3_ is thermally unstable, it is likely that unreacted material decomposed under electron beam exposure in the SEM, resulting in Sb residues as the chlorine components evaporated.

At the end of the reaction, the amounts of C, H, N, S, and O decreased due to the decomposition of cysteine starting around 200 °C.^73^ The main compartments of the residues are N, C, and H. The amount of Cl did not change. It seems to form a stable compound part of the matrix.

Thus, the matrix appears to be a compound derived from cysteine, ODE, and chlorine that decomposes to a different composition upon heating.

## Conclusions

In summary, a one-pot synthesis for the preparation of spherical crystalline sub-micron Sb_2_S_3_ particles has been established. In ODE, the insoluble precursors, SbCl_3_ and cysteine, form an intermediate upon heating. In addition, excess cysteine, ODE, and chloride from SbCl_3_ form a matrix that encapsulates this intermediate and prevents the initially resulting amorphous particles from aggregating and restructuring into large rod-like shapes upon crystallization. The matrix is removable and the purified particles are stable in dispersion.

It was shown that the intermediate of the particles is likely to consist of a complex architecture with a high degree of crosslinking. The basic building block of the intermediate is assumed to be an Sb atom bound to three cysteine molecules *via* a covalent Sb–S bond. These units are connected by coordinative bonds between Sb and the other functional groups of the cysteine molecules.

The matrix is formed from cysteine, the solvent ODE, and chlorine from the SbCl_3_. Due to partial decomposition, the composition of the matrix changes during the heat-up process. It may be worthwhile to try mixing in another amino acid, such as glycine, and examine the effect on the particle size and shape.

The concept of encapsulation for size and/or shape control of colloid particles could be transferrable to other materials. Depending on the particle system, it may allow an extension of the size range to smaller particles and force nearly isotropic growth for anisotropic materials. In such approaches, the use of cysteine or cysteine–amino acid mixtures may be applicable to various other sulfide materials.^74^

## Author contributions

M. Joschko: design and development of the synthesis, characterization of the samples, and preparation of the original draft. C. Malsi: design and development of the synthesis. P. Scharmann and J. Rapier: optimization of the synthesis. S. Selve: TEM measurements. C. Graf: acquisition of funding, supervision, project management, review, and editing of the manuscript.

## Conflicts of interest

There are no conflicts to declare.

## Supplementary Material

NA-006-D4NA00020J-s001

## References

[cit1] Gruber H. (1992). The learning curve in the production of semiconductor memory chips. Appl. Econ..

[cit2] Hu X., Krull P., de Graff B., Dowling K., Rogers J. A., Arora W. J. (2011). Stretchable inorganic-semiconductor electronic systems. Adv. Mater..

[cit3] Tricoli A., Righettoni M., Teleki A. (2010). Semiconductor gas sensors: dry synthesis and application. Angew. Chem., Int. Ed..

[cit4] Wang L., Zhao J., Liu H., Huang J. (2018). Design, modification and application of semiconductor photocatalysts. J. Taiwan Inst. Chem. Eng..

[cit5] Martynenko I. V., Litvin A. P., Purcell-Milton F., Baranov A. V., Fedorov A. V., Gun’ko Y. K. (2017). Application of semiconductor quantum dots in bioimaging and biosensing. J. Mater. Chem. B.

[cit6] Peter L. M. (2011). Towards sustainable photovoltaics: the search for new materials. Philos. Trans. R. Soc., A.

[cit7] Cao F., Liu W., Zhou L., Deng R., Song S., Wang S., Su S., Zhang H. (2011). Well-defined Sb_2_S_3_ microspheres: high-yield synthesis, characterization, their optical and electrochemical hydrogen storage properties. Solid State Sci..

[cit8] Shaji S., Arato A., O'Brien J. J., Liu J., Castillo G. A., Palma M. I. M., Roy T. K. D., Krishnan B. (2010). Chemically deposited Sb_2_S_3_ thin films for optical recording. J. Phys. D: Appl. Phys..

[cit9] Tang R., Wang X., Jiang C., Li S., Jiang G., Yang S., Zhu C., Chen T. (2018). Vacuum assisted solution processing for highly efficient Sb_2_S_3_ solar cells. J. Mater. Chem. A.

[cit10] Wang X., Tang R., Wu C., Zhu C., Chen T. (2018). Development of antimony sulfide–selenide Sb_2_(S, Se)_3_-based solar cells. J. Energy Chem..

[cit11] Validžić I. L., Janošević V., Mitrić M. (2016). Characterization and current-voltage characteristics of solar cells based on the composite of synthesized Sb_2_S_3_ powder with small band gap and natural dye. Environ. Prog. Sustainable Energy.

[cit12] Versavel M. Y., Haber J. A. (2007). Structural and optical properties of amorphous and crystalline antimony sulfide thin-films. Thin Solid Films.

[cit13] Sahoo R. K., Singh S., Yun J. M., Kwon S. H., Kim K. H. (2019). Sb_2_S_3_ Nanoparticles Anchored or Encapsulated by the Sulfur-Doped Carbon Sheet for High-Performance Supercapacitors. ACS Appl. Mater. Interfaces.

[cit14] Zhang Q., Zeng Y., Wang X., Wang L., Wang H., Xiao J., Li X. (2021). Sb_2_S_3_ nanoparticles anchored on N-doped 3D carbon nanofibers as anode material for sodium ion batteries with improved electrochemical performance. J. Alloys Compd..

[cit15] Farhana M. A., Manjceevan A., Bandara J. (2023). Recent advances and new research trends in Sb_2_S_3_ thin film based solar cells. J. Sci.: Adv. Mater. Devices.

[cit16] Li X., Bai J., Zhou B., Yuan X., Zhang X., Liu L. (2018). High Performance of 3D Symmetric Flowerlike Sb_2_S_3_ Nanostructures in Dye-Sensitized Solar Cells. Chem.–Eur. J..

[cit17] Wang W., Strössner F., Zimmermann E., Schmidt-Mende L. (2017). Hybrid solar cells from Sb_2_S_3_ nanoparticle ink. Sol. Energy Mater. Sol. Cells.

[cit18] Hosono H. (2007). 68.3: Invited Paper: Transparent Amorphous Oxide Semiconductors for High Performance TFT. SID Int. Symp. Dig. Tech. Pap..

[cit19] Alves H., Pinto R. M., Maçôas E. S. (2013). Photoconductive response in organic charge transfer interfaces with high quantum efficiency. Nat. Commun..

[cit20] Lou W., Chen M., Wang X., Liu W. (2007). Novel Single-Source Precursors Approach to Prepare Highly Uniform Bi_2_S3 and Sb_2_S_3_ Nanorods *via* a Solvothermal Treatment. Chem. Mater..

[cit21] Ota J., Roy P., Srivastava S. K., Nayak B. B., Saxena A. K. (2008). Morphology Evolution of Sb_2_S_3_ under Hydrothermal Conditions: Flowerlike Structure to Nanorods. Cryst. Growth Des..

[cit22] Salinas-Estevané P., Sánchez E. M. (2010). Preparation of Sb_2_S_3_ Nanostructures by the Ionic Liquid-Assisted Sonochemical Method. Cryst. Growth Des..

[cit23] Salem A. M., Selim M. S. (2001). Structure and optical properties of chemically deposited Sb_2_S_3_ thin films. J. Phys. D: Appl. Phys..

[cit24] Murtaza G., Akhtar M., Azad Malik M., O'Brien P., Revaprasadu N. (2015). Aerosol assisted chemical vapor deposition of Sb_2_S_3_ thin films: environmentally benign solar energy material. Mater. Sci. Semicond. Process..

[cit25] Dutta A. K., Maji S. K., Mitra K., Sarkar A., Saha N., Ghosh A. B., Adhikary B. (2014). Single source precursor approach to the synthesis of Bi_2_S_3_ nanoparticles: a new amperometric hydrogen peroxide biosensor. Sens. Actuators, B.

[cit26] Piras R., Aresti M., Saba M., Marongiu D., Mula G., Quochi F., Mura A., Cannas C., Mureddu M., Ardu A., Ennas G., Calzia V., Mattoni A., Musinu A., Bongiovanni G. (2014). Colloidal synthesis and characterization of Bi_2_S_3_ nanoparticles for photovoltaic applications. J. Phys.: Conf. Ser..

[cit27] Uddin I., Abzal S. M., Kalyan K., Janga S., Rath A., Patel R., Gupta D. K., Ravindran T. R., Ateeq H., Khan M. S., Dash J. K. (2022). Starch-Assisted Synthesis of Bi_2_S_3_ Nanoparticles for Enhanced Dielectric and Antibacterial Applications. ACS Omega.

[cit28] Abulikemu M., Del Gobbo S., Anjum D. H., Malik M. A., Bakr O. M. (2016). Colloidal Sb_2_S_3_ nanocrystals: synthesis, characterization and fabrication of solid-state semiconductor sensitized solar cells. J. Mater. Chem. A.

[cit29] Joschko M., Fotue Wafo F. Y., Malsi C., Kisić D., Validžić I., Graf C. (2021). Revealing the formation mechanism and band gap tuning of Sb_2_S_3_ nanoparticles. Beilstein J. Nanotechnol..

[cit30] Maiti N., Im S. H., Lee Y. H., Seok S. I. (2012). Urchinlike nanostructure of single-crystalline nanorods of Sb_2_S_3_ formed at mild reaction condition. ACS Appl. Mater. Interfaces.

[cit31] Chao J., Liang B., Hou X., Liu Z., Xie Z., Liu B., Song W., Chen G., Chen D., Shen G. (2013). Selective synthesis of Sb_2_S_3_ nanoneedles and nanoflowers for high performance rigid and flexible photodetectors. Opt. Express.

[cit32] Ma J., Duan X., Lian J., Kim T., Peng P., Liu X., Liu Z., Li H., Zheng W. (2010). Sb_2_S_3_ with various nanostructures: controllable synthesis, formation mechanism, and electrochemical performance toward lithium storage. Chem.–Eur. J..

[cit33] Kravchyk K. V., Kovalenko M. V., Bodnarchuk M. I. (2020). Colloidal Antimony Sulfide Nanoparticles as a High-Performance Anode Material for Li-ion and Na-ion Batteries. Sci. Rep..

[cit34] Wang H., Lu Y.-N., Zhu J.-J., Chen H.-Y. (2003). Sonochemical fabrication and characterization of stibnite nanorods. Inorg. Chem..

[cit35] House A., Kuna A., Hastings D., Rodriguez N., Schoenitz M., Dreizin E. L., Guvendiren M. (2023). Effect of particle shape on rheology and printability of highly filled reactive inks for direct ink writing. Prog. Addit. Manuf..

[cit36] Zhang L., Feng G., Zeravcic Z., Brugarolas T., Liu A. J., Lee D. (2013). Using shape anisotropy to toughen disordered nanoparticle assemblies. ACS Nano.

[cit37] Enríquez E., Reinosa J. J., Fuertes V., Fernández J. F. (2022). Advances and challenges of ceramic pigments for inkjet printing. Ceram. Int..

[cit38] Huang Y., Jiang L., Li B., Premaratne P., Jiang S., Qin H. (2020). Study effects of particle size in metal nanoink for electrohydrodynamic inkjet printing through analysis of droplet impact behaviors. J. Manuf. Process..

[cit39] Wei Q., Chang D., Ye Z., Li X., Zan L., Gao L., Fu F., Yang D. (2021). Giant improvement of performances of perovskite solar cells *via* component engineering. J. Colloid Interface Sci..

[cit40] Kamiya H. , Gotoh K., Shimada M., Uchikoshi T., Otani Y., Fuji M., Matsusaka S., Matsuyama T., Tatami J., Higashitani K., Kurihara K., Ishida N., Suzuki M., Abe H., Otsubo Y. and Miyahara M., Characteristics and Behavior of Nanoparticles and its Dispersion Systems, Elsevier, Amsterdam, 2008

[cit41] Baalousha M., Manciulea A., Cumberland S., Kendall K., Lead J. R. (2008). Aggregation and surface properties of iron oxide nanoparticles: influence of pH and natural organic matter. Environ. Toxicol. Chem..

[cit42] Somosi Z., Pavlovic M., Pálinkó I., Szilágyi I. (2018). Effect of Polyelectrolyte Mono- and Bilayer Formation on the Colloidal Stability of Layered Double Hydroxide Nanoparticles. Nanomater.

[cit43] Zhu Y., Jiang F. Y., Chen K., Kang F., Tang Z. K. (2011). Size-controlled synthesis of monodisperse superparamagnetic iron oxide nanoparticles. J. Alloys Compd..

[cit44] Park J., An K., Hwang Y., Park J.-G., Noh H.-J., Kim J.-Y., Park J.-H., Hwang N.-M., Hyeon T. (2004). Ultra-large-scale syntheses of monodisperse nanocrystals. Nat. Mater..

[cit45] Pawlik V., Zhou S., Zhou S., Qin D., Xia Y. (2023). Silver Nanocubes: From Serendipity to Mechanistic Understanding, Rational Synthesis, and Niche Applications. Chem. Mater..

[cit46] Eisa W. H., Abdel-Moneam Y. K., Shaaban Y., Abdel-Fattah A. A., Abou Zeid A. M. (2011). Gamma-irradiation assisted seeded growth of Ag nanoparticles within PVA matrix. Mater. Chem. Phys..

[cit47] Alexandrov A., Smirnova L., Yakimovich N., Sapogova N., Soustov L., Kirsanov A., Bityurin N. (2005). UV-initiated growth of gold nanoparticles in PMMA matrix. Appl. Surf. Sci..

[cit48] Di Luccio T., Laera A. M., Tapfer L., Kempter S., Kraus R., Nickel B. (2006). Controlled nucleation and growth of CdS nanoparticles in a polymer matrix. J. Phys. Chem. B.

[cit49] Ibáñez J., Sans J. A., Popescu C., López-Vidrier J., Elvira-Betanzos J. J., Cuenca-Gotor V. P., Gomis O., Manjón F. J., Rodríguez-Hernández P., Muñoz A. (2016). Structural, Vibrational, and Electronic Study of Sb_2_S_3_ at High Pressure. J. Phys. Chem. C.

[cit50] Zhu Y. F., Fan D. H., Shen W. Z. (2008). Chemical conversion synthesis and optical properties of metal sulfide hollow microspheres. Langmuir.

[cit51] Pan J., Xiong S., Xi B., Li J., Li J., Zhou H., Qian Y. (2009). Tartatric Acid and L-Cysteine Synergistic-Assisted Synthesis of Antimony Trisulfide Hierarchical Structures in Aqueous Solution. Eur. J. Inorg. Chem..

[cit52] Viezbicke B. D., Patel S., Davis B. E., Birnie D. P. (2015). Evaluation of the Tauc method for optical absorption edge determination: ZnO thin films as a model system. Phys. Status Solidi B.

[cit53] Schindelin J., Arganda-Carreras I., Frise E., Kaynig V., Longair M., Pietzsch T., Preibisch S., Rueden C., Saalfeld S., Schmid B., Tinevez J.-Y., White D. J., Hartenstein V., Eliceiri K., Tomancak P., Cardona A. (2012). Fiji: an open-source platform for biological-image analysis. Nat. Methods.

[cit54] Holzwarth U., Gibson N. (2011). The Scherrer equation *versus* the ‘Debye-Scherrer equation’. Nat. Nanotechnol..

[cit55] Kubelka P., Munk F. (1931). An Article on Optics of Paint Layers. Z. Tech. Phys..

[cit56] Tauc J., Grigorovici R., Vancu A. (1966). Optical Properties and Electronic Structure of Amorphous Germanium. Phys. Status Solidi B.

[cit57] Davis E. A., Mott N. F. (1970). Conduction in non-crystalline systems V. Conductivity, optical absorption and photoconductivity in amorphous semiconductors. Philos. Mag..

[cit58] Lei H., Lin T., Wang X., Zhang S., Cheng Q., Chen X., Tan Z., Chen J. (2018). A novel *in situ* hydrothermal preparation route for Sb_2_S_3_ and its solar cell application. Mater. Lett..

[cit59] Wang G., Cheung C. L. (2012). Building crystalline Sb_2_S_3_ nanowire dandelions with multiple crystal splitting motif. Mater. Lett..

[cit60] Han Q., Sun S., Sun D., Zhu J., Wang X. (2011). Room-temperature synthesis from molecular precursors and photocatalytic activities of ultralong Sb_2_S_3_ nanowires. RSC Adv..

[cit61] Gao C., Huang J., Li H., Sun K., Lai Y., Jia M., Jiang L., Liu F. (2019). Fabrication of Sb_2_S_3_ thin films by sputtering and post-annealing for solar cells. Ceram. Int..

[cit62] Perales F., Lifante G., Agulló-Rueda F., Heras C. d. l. (2007). Optical and structural properties in the amorphous to polycrystalline transition in Sb_2_S_3_ thin films. J. Phys. D: Appl. Phys..

[cit63] Ţigau N., Gheorghieş C., Rusu G. I., Condurache-Bota S. (2005). The influence of the post-deposition treatment on some physical properties of Sb_2_S_3_ thin films. J. Non-Cryst. Solids.

[cit64] Vadapoo R., Krishnan S., Yilmaz H., Marin C. (2011). Self-standing nanoribbons of antimony selenide and antimony sulfide with well-defined size and band gap. Nanotechnology.

[cit65] Filip M. R., Patrick C. E., Giustino F. (2013). GW quasiparticle band structures of stibnite, antimonselite, bismuthinite, and guanajuatite. Phys. Rev. B: Condens. Matter Mater. Phys..

[cit66] Su Y., Hessou E. P., Colombo E., Belletti G., Moussadik A., Lucas I. T., Frochot V., Daudon M., Rouzière S., Bazin D., Li K., Quaino P., Tielens F. (2022). Crystalline structures of L-cysteine and L-cystine: a combined theoretical and experimental characterization. Amino acids.

[cit67] Urgut O. S., Ozturk I. I., Banti C. N., Kourkoumelis N., Manoli M., Tasiopoulos A. J., Hadjikakou S. K. (2016). Addition of tetraethylthiuram disulfide to antimony(III) iodide; synthesis, characterization and biological activity. Inorg. Chim. Acta.

[cit68] Tossell J. A. (1994). The speciation of antimony in sulfidic solutions: a theoretical study. Geochim. Cosmochim. Acta.

[cit69] Wesdemiotis C., Williams-Pavlantos K. N., Keating A. R., McGee A. S., Bochenek C. (2024). Mass spectrometry of polymers: a tutorial review. Mass Spectrom. Rev..

[cit70] Dostál L., Jambor R., Růžička A., Lyčka A., Brus J., de Proft F. (2008). Synthesis and Structure of Organoantimony(III) Compounds Containing Antimony−Selenium and −Tellurium Terminal Bonds. Organometallics.

[cit71] Ozturk I. I., Banti C. N., Kourkoumelis N., Manos M. J., Tasiopoulos A. J., Owczarzak A. M., Kubicki M., Hadjikakou S. K. (2014). Synthesis, characterization and biological activity of antimony(III) or bismuth(III) chloride complexes with dithiocarbamate ligands derived from thiuram degradation. Polyhedron.

[cit72] Tella M., Pokrovski G. S. (2009). Antimony(III) complexing with O-bearing organic ligands in aqueous solution: an X-ray absorption fine structure spectroscopy and solubility study. Geochim. Cosmochim. Acta.

[cit73] Weiss I. M., Muth C., Drumm R., Kirchner H. O. K. (2018). Thermal decomposition of the amino acids glycine, cysteine, aspartic acid, asparagine, glutamic acid, glutamine, arginine and histidine. BMC Biophys..

[cit74] Jamal F., Rafique A., Moeen S., Haider J., Nabgan W., Haider A., Imran M., Nazir G., Alhassan M., Ikram M., Khan Q., Ali G., Khan M., Ahmad W., Maqbool M. (2023). Review of Metal Sulfide Nanostructures and their Applications. ACS Appl. Nano Mater..

