# Supplementary material for: Colloidal spherical stibnite particles via high-temperature metallo-organic synthesis
Source: Nanoscale Adv. 2024 Jul 11;6(17):4450–61. doi: 10.1039/d4na00020j (PMC11334972; doi:10.1039/d4na00020j)
Supplement: NA-006-D4NA00020J-s001 [file NA-006-D4NA00020J-s001.pdf]

Electronic Supplementary Material (ESI) for Nanoscale Advances.
This journal is © The Royal Society of Chemistry 2024

Electronic supplementary information (ESI)

Colloidal spherical stibnite particles via high-temperature metallo-organic synthesis

Maximilian Joschko[a], Christina Malsi[a], John Rapier[a], Paolo Scharmann[a], Sören Selve[b], and Christina Graf*[a]

[a]Hochschule Darmstadt - University of Applied Sciences, Fachbereich Chemie- und Biotechnologie, Stephanstr. 7, D-64295 Darmstadt, Germany

[b]Technische Universität Berlin, Zentraleinrichtung Elektronenmikroskopie (ZELMI), Straße des 17. Juni 135, D-10623 Berlin, Germany

**Corresponding author | Prof. Dr. Christina Graf | christina.graf@h-da.de*

Particle purification

The as-received particles were purified by subsequent centrifugation and redispersing in different solvents. The solvents used during the purification process were isopropyl alcohol (IPA), water, and dimethyl formamide (DMF).
First, the reaction mixture was divided into two 50 mL centrifuge tubes of 12.5 mL each and was made up to 40 mL with IPA. The tubes were sonicated for 5 min using an ultrasonic bath for 5 min. The particles were then centrifuged at 1000 rcf for 20 min, and the supernatant was removed. Subsequently, the particles were redispersed in a water/IPA mixture using an ultrasonic processor (UP50H, Hielscher; 1 cycle, 100 % amplitude, tip: MS3). The following purification steps were performed analogously and are summarized in Table S1. Finally, the particles were redispersed in 5 mL H_2O.

Table S1 Particle purification steps 2-7.

Step	**Solvent**	**Ultrasonic treatment (min)**	**Speed (centrifuge) (rcf)**	**Duration (centrifuge) (min)**
2	20 mL H_2O<br>20 mL IPA	20	1500	20
3	20 mL H_2O<br>20 mL IPA	20	1500	20
4	40 mL DMF	20	1500	20
5	40 mL DMF	20	1500	20
6	20 mL H_2O<br>20 mL IPA	20	1500	20
7	40 mL H_2O	10	1500	20

Additional measurement data:

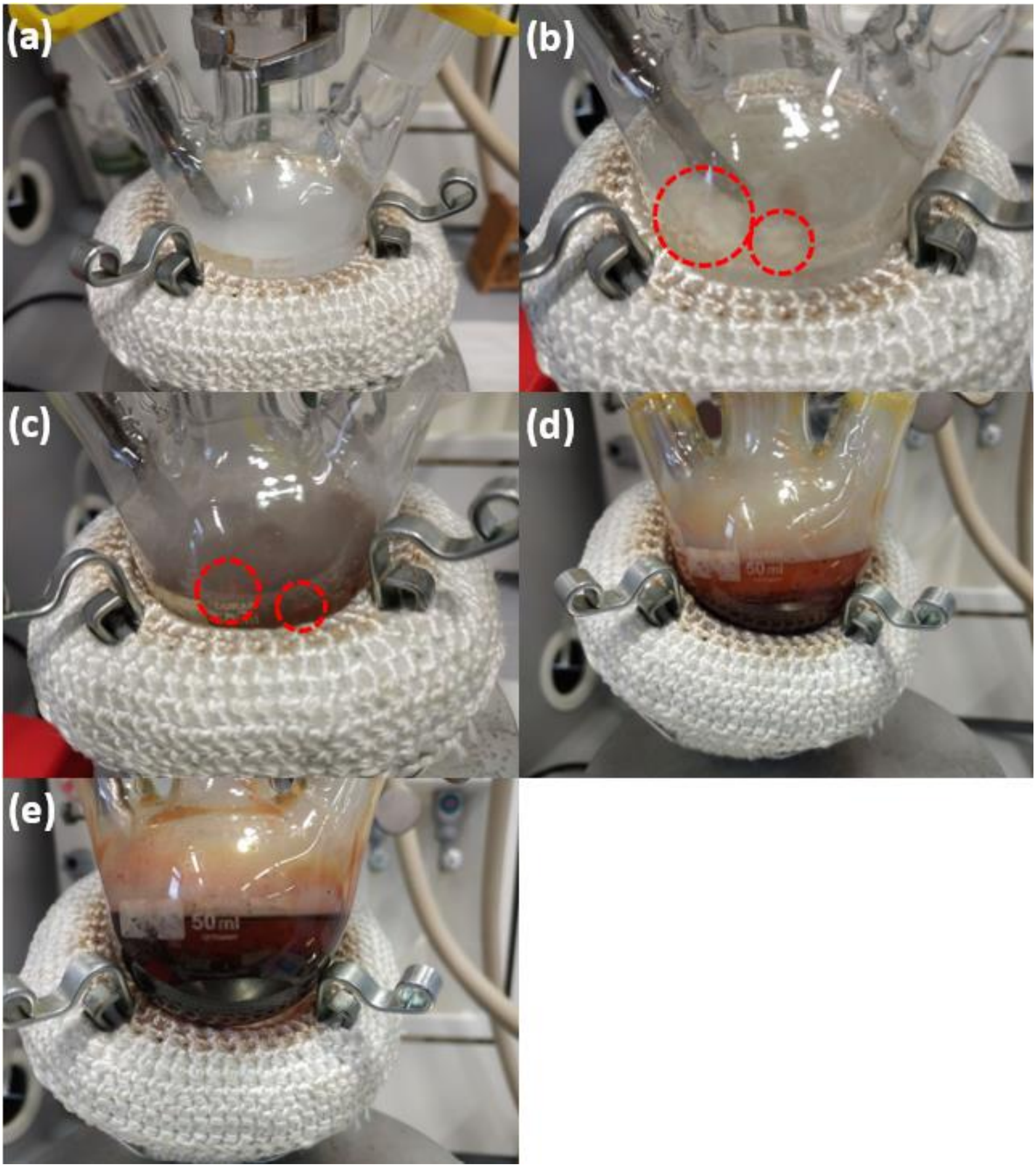

Fig. S1 Photographs of the reaction mixture at different reaction stages: (a) dispersed precursors at ambient temperature (P0), (b) reaction at 140 °C (PI), (c) reaction at 170 °C (PII), (d) reaction at 200 °C (PIII), and (e) reaction at 240 °C (PIV). The red dotted circles indicate flocculated intermediate.

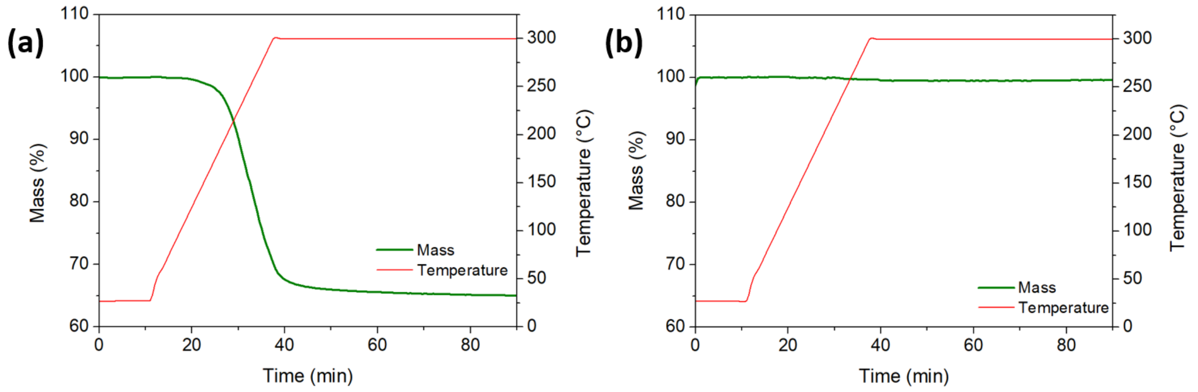

Fig. S2 TGA graphs showing the changes in mass for a given temperature profile of (a) a sample with matrix around the nanoparticles and (b) a purified sample. The small positive drift in (b) is most likely due to the instrument itself.

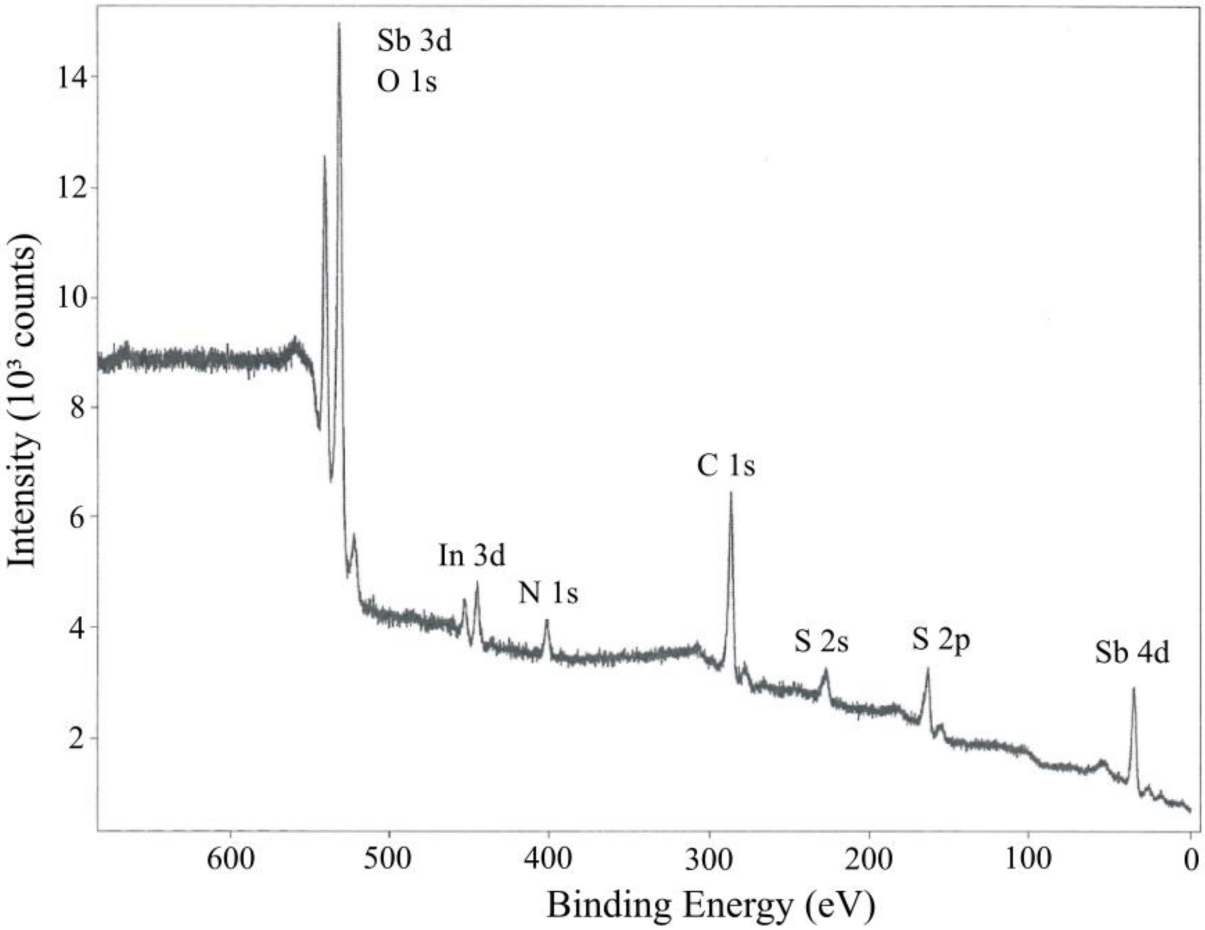

Fig. S3 XPS data of the purified final sample of Sb_2S_3 particles. The In 3d peak is found due to the indium foil in which the sample was wrapped.

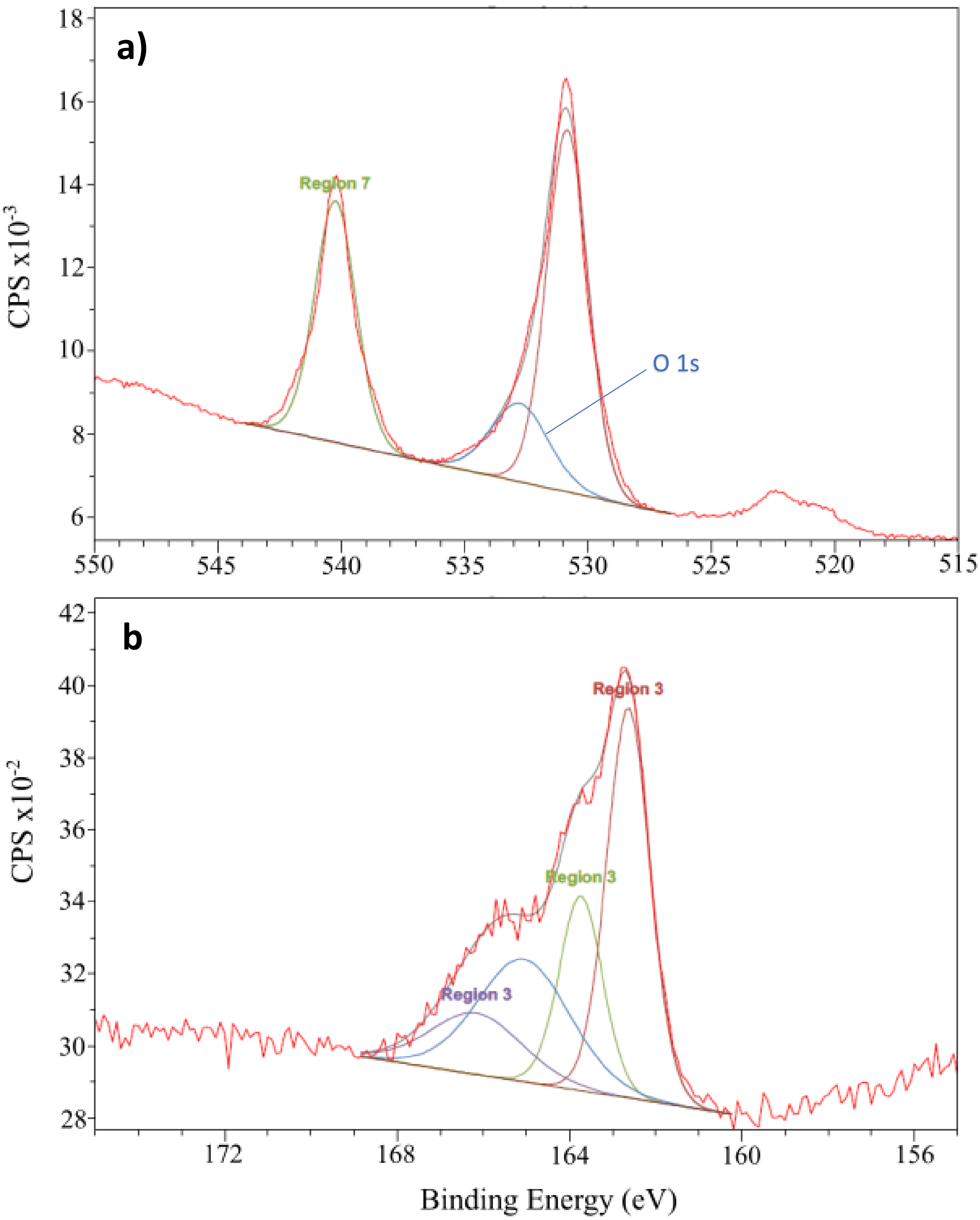

Fig. S4 Detailed XPS analysis of Sb 3d/O 1s (a) and S 2p (b) in the purified final sample.

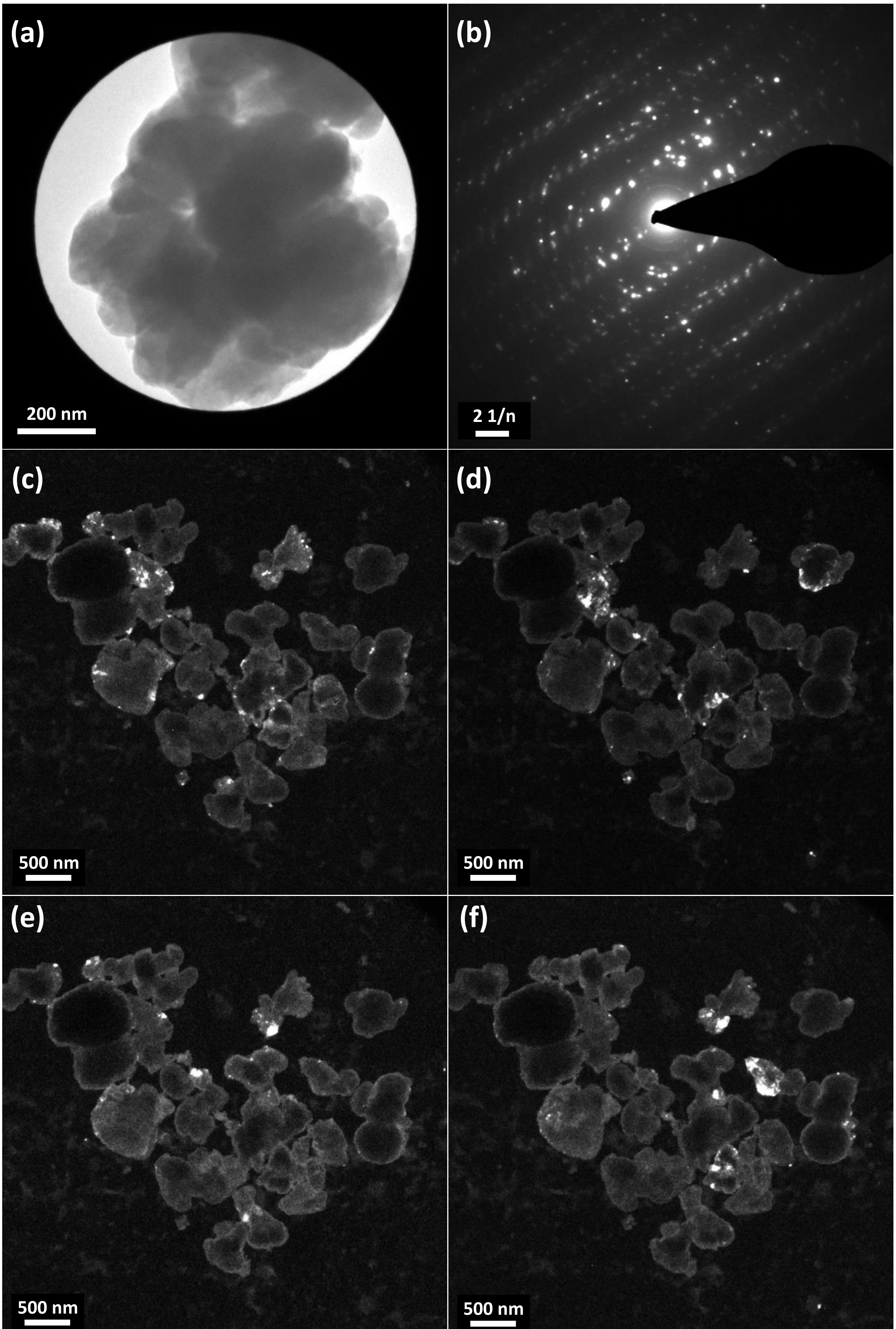

Fig. S5 (a) TEM image of a particle with (b) the corresponding SAED pattern, and (c) to (f) series of dark-field images of several particles showing different crystal planes in different observation planes.

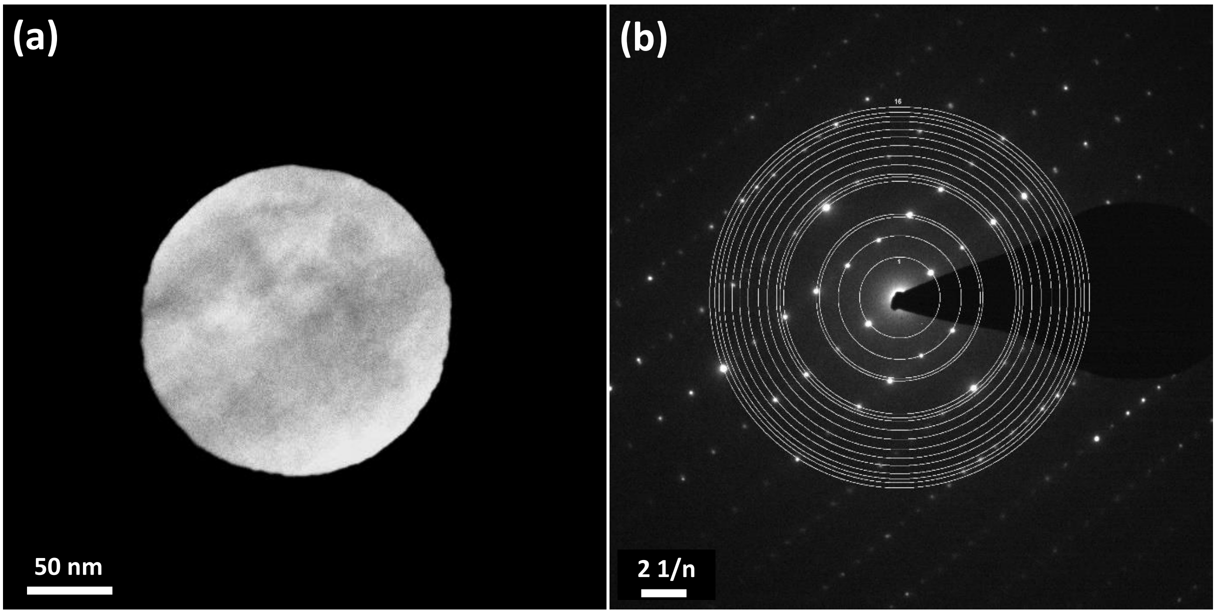

Fig. S6 (a) HRTEM image of a section of a stibnite particle and (b) the corresponding SAED image with analysis of the reflexes.

Table S2 Evaluation data taken from the SAED analysis: the diameter of the SAED rings D, the calculated interplanar spacing d as well as its reference data d_{ref}, and the corresponding hkl indices.

Reflex	**D (1/nm)**	**d (Å) ±0.05 Å**	**d_{ref} (Å)^a**	**hkl**
1	3.5704	5.602	5.625	200
2	5.4595	3.663	3.631	101
3	7.1219	2.808	2.765	221
4	7.3738	2.712	2.725	410
5	10.2704	1.947	1.947	341
6	10.6230	1.883	1.886	060
7	10.9001	1.835	1.842	251
8	11.7061	1.709	1.693	061
9	12.5121	1.598	1.600	541
10	13.4189	1.490	1.484	370
11	14.1745	1.411	1.414	522
12	14.8294	1.349	1.352	561
13	15.5347	1.287	1.292	281
14	15.9881	1.251	1.245	831
15	16.3407	1.224	1.225	712
16	16.7941	1.191	1.200	481

[a]References are taken from JCPDS card 42-1393

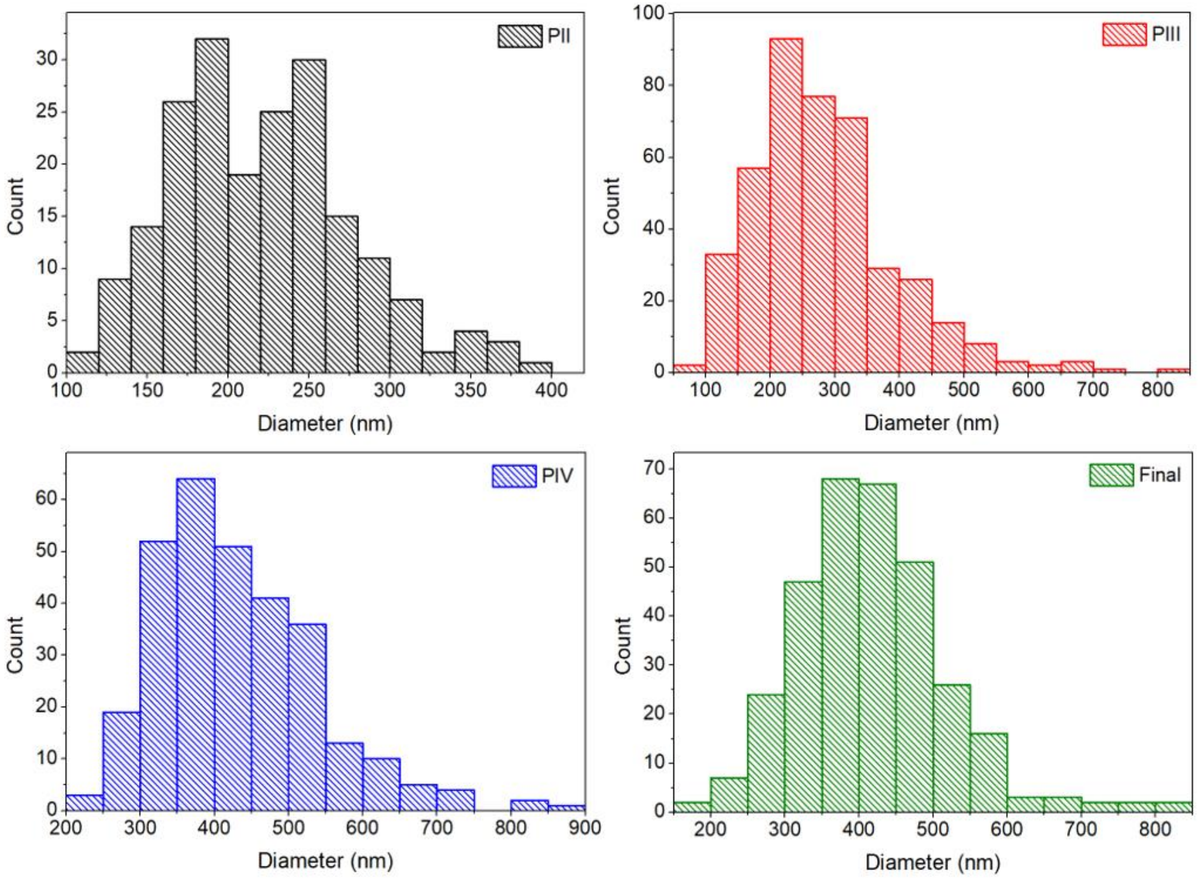

Fig. S7 Histograms showing the size distribution of the Sb_2S_3 particles across the different reaction stages.

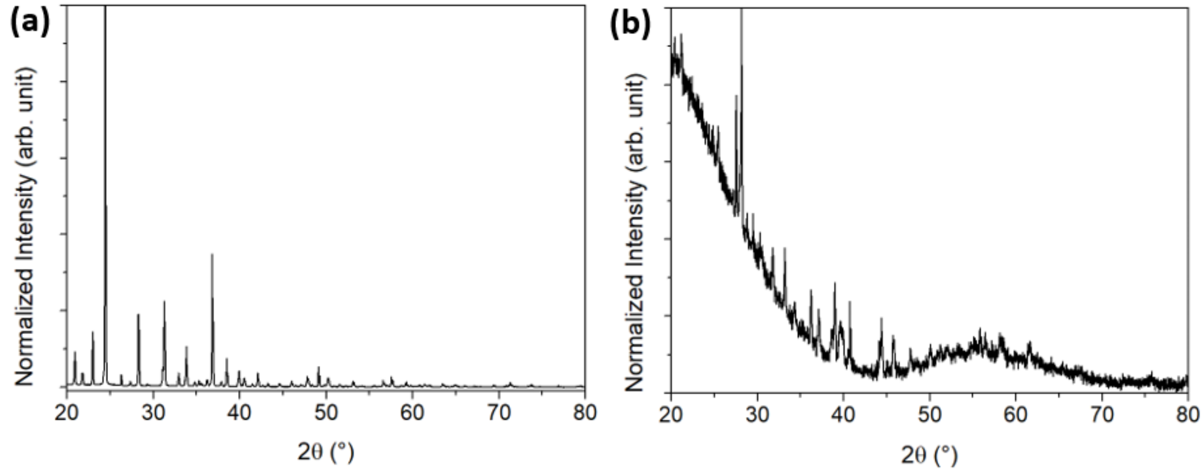

Fig. S8 Reference X ray diffractograms of (a) L-cysteine and (b) $SbCl_3$. The high background signal in (b) is occurring due to the strong hygroscopy of the material.

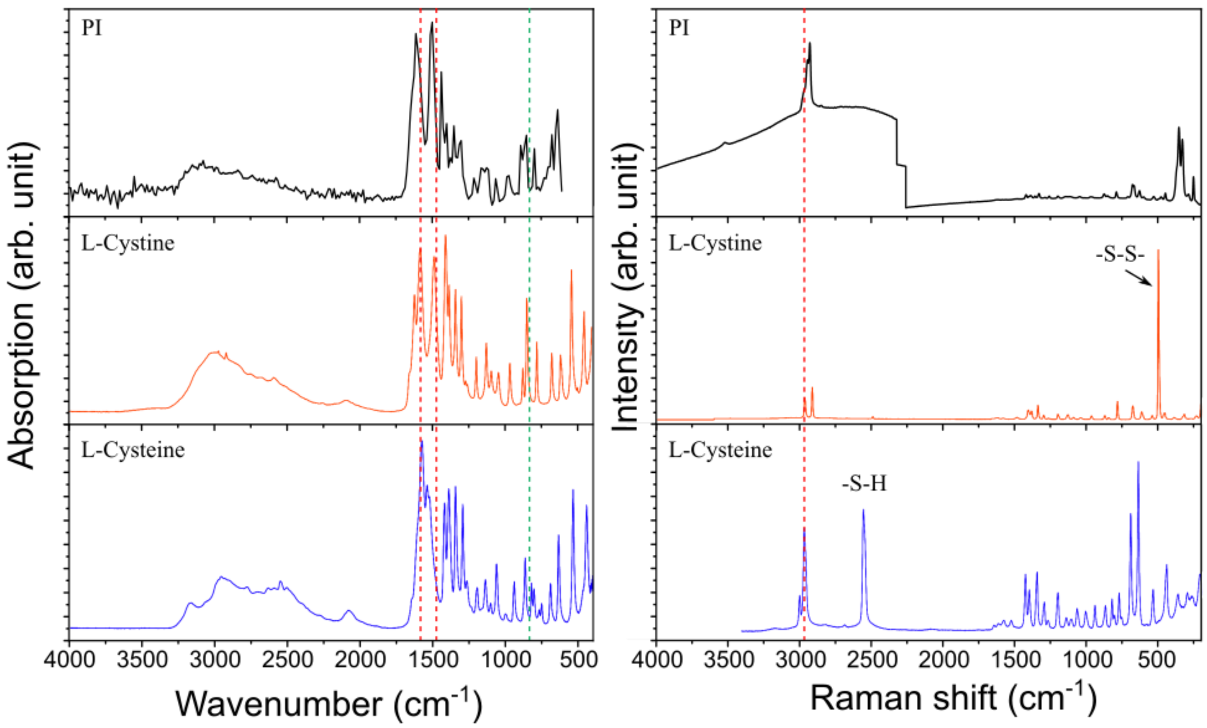

Fig. S9 FTIR (right) and Raman (left) spectra of the sample PI (140 °C) as well as L-cysteine and L-cystine as a reference.[1] The dotted lines help to localize the peak values.

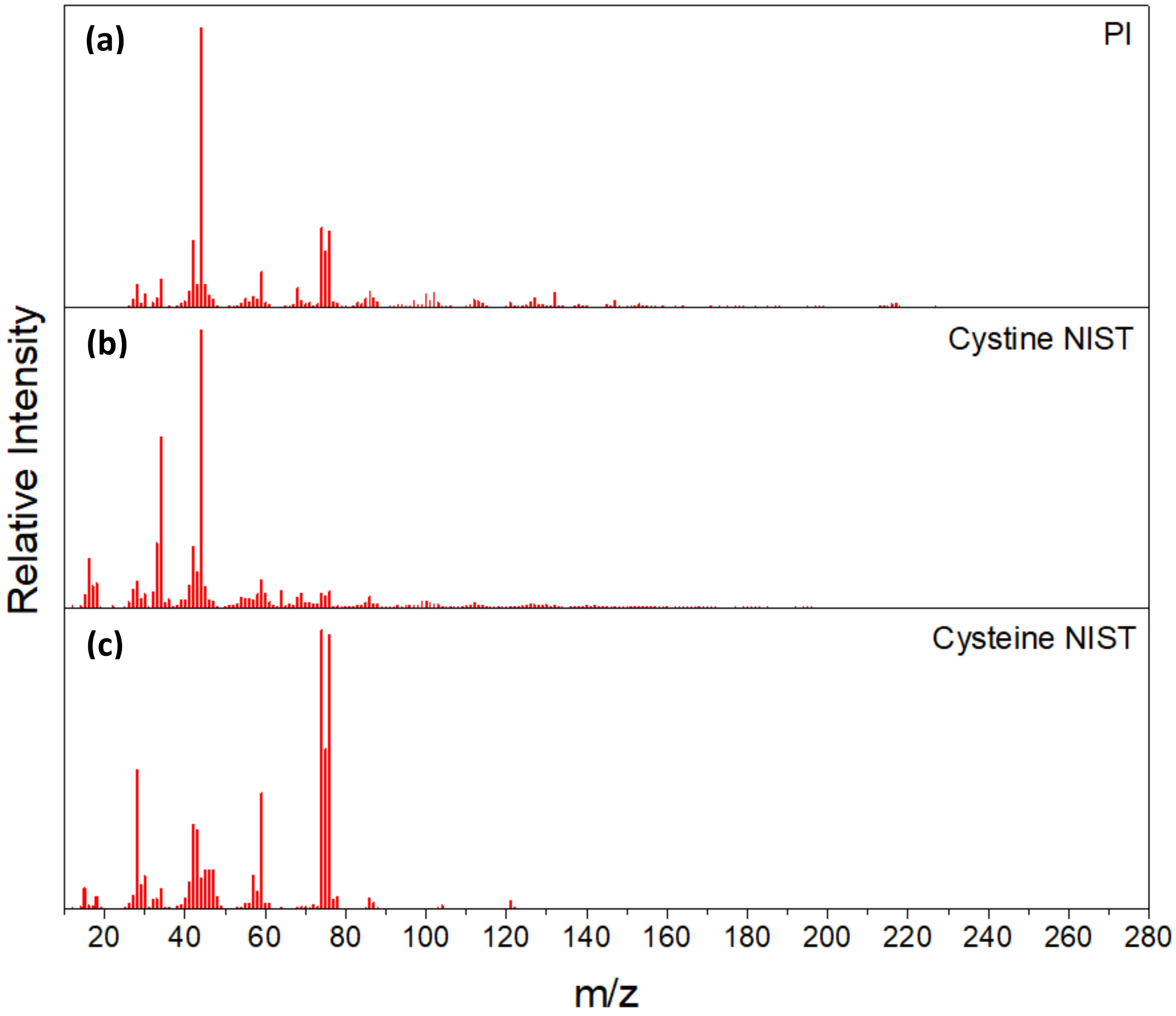

Fig. S10 EI mass spectrum of the sample PI in comparison with the reference data of cystine and cysteine from the National Institute of Standards and Technology (NIST) database (MS numbers 228153 and 228086, respectively).[2]

References

1 John Wiley & Sons, Inc. SpectraBase; https://spectrabase.com/ (retrieved August 14, 2023)

2 William E. Wallace, *NIST Mass Spectrometry Data Center, "Mass Spectra" in NIST Chemistry WebBook, NIST Standard Reference Database Number 69*, Eds. P.J. Linstrom and W.G. Mallard, National Institute of Standards and Technology, Gaithersburg MD, 20899, (retrieved August 14, 2023)
